# IoT Sensing for Advanced Irrigation Management: A Systematic Review of Trends, Challenges, and Future Prospects

**DOI:** 10.3390/s25072291

**Published:** 2025-04-04

**Authors:** Ahmed A. Abdelmoneim, Hilda N. Kimaita, Christa M. Al Kalaany, Bilal Derardja, Giovanna Dragonetti, Roula Khadra

**Affiliations:** Mediterranean Agronomic Institute CIHEAM Bari, Valenzano, 70010 Bari, Italy; ayoub@iamb.it (A.A.A.); kimaitahilda@gmail.com (H.N.K.); christamaria.kalaany@std.balamand.edu.lb (C.M.A.K.); derardja@iamb.it (B.D.); dragonetti@iamb.it (G.D.)

**Keywords:** internet of things (IoT), smart agriculture, soil moisture, wireless sensor networks (WSN), digital agriculture, bibliometric analysis

## Abstract

Efficient water management is crucial for sustainable agriculture, and the integration of Internet of Things (IoT) technologies in irrigation systems offers innovative solutions to optimize resource use. In this systematic review, the current landscape of Internet of Things (IoT) applications in irrigation management was investigated. The study aimed to identify key research trends and technological developments in the field. Using VOSviewer (CWTS, Leiden, The Netherlands) for bibliometric mapping, the influential research clusters were identified. The analysis revealed a significant rise in scholarly interest, with peak activity between 2020 and 2022, and a shift towards interdisciplinary and applied research. Additionally, the content analysis revealed prevalent agricultural applications, frequently employed microcontroller units (MCUs), widely used sensors, and trends in communication technologies such as the increasing adoption of low-power, scalable communication protocols for real-time data acquisition. This study not only offers a comprehensive overview of the current status of IoT integration in smart irrigation but also highlights the technological advancements. Future research directions include integrating IoT with emerging technologies such as artificial intelligence, edge computing, and blockchain to enhance decision-support systems and predictive irrigation strategies. By examining the transformative potential of IoT, this study provides valuable insights for researchers and practitioners seeking to enhance agricultural productivity, optimize resource use, and improve sustainability.

## 1. Introduction

The integration of the Internet of Things (IoT) in agricultural applications has emerged as a transformative force in modern agriculture, fostering smarter and more-efficient agricultural practices. This shift is driven by advancements in electronics, data science, and communication technologies, which have catalyzed the evolution from traditional farming to precision and smart agriculture [1].

IoT in agriculture involves deploying interconnected sensors and devices that gather real-time data on soil conditions, crop health, weather, and environmental parameters [2]. Recent market analyses indicate a robust growth trajectory for the Internet of Things (IoT) in the agricultural sector. The global agriculture IoT market is projected to expand from USD 11.4 billion in 2021 to USD 18.1 billion by 2026, reflecting a compound annual growth rate (CAGR) of 9.8% during this period [3]. Similarly, [4] forecasts that the IoT in the agriculture market will grow from USD 16.24 billion in 2024 to approximately USD 40.24 billion by 2034, with a CAGR of 9.5% between 2024 and 2034. These projections underscore the increasing adoption of IoT technologies in agriculture, driven by the need for enhanced productivity, resource efficiency, and sustainable farming practices.

Globally, irrigated agriculture accounts for 70% of freshwater consumption, making it both a primary driver and a direct consequence of water scarcity. As a result, enhancing on-farm irrigation management is crucial [5]. One of the key factors that can significantly improve irrigation management is the implementation of reliable IoT data acquisition systems [6]. These systems provide real-time insights into soil moisture levels, weather conditions, and plant health, forming the foundation of precision irrigation by enabling timely and informed decision-making. The integration of Internet of Things (IoT) technology into irrigation management offers an innovative approach to resource optimization, particularly through smart irrigation systems. While the Internet of Things (IoT) holds significant promise for revolutionizing agricultural irrigation, several challenges impede its widespread adoption. Data privacy and security concerns are paramount, as unauthorized breaches and cyber-attacks on data collected from IoT devices pose significant risks [7]. Additionally, the lack of reliable Internet infrastructure in rural areas hinders the seamless integration of IoT technologies [8]. However, as IoT technology continues to mature and costs decrease, its adoption is expected to become more widespread, transforming agriculture into a data-driven, sustainable, and efficient sector capable of meeting the demands of a growing global population.

**Figure 1:**
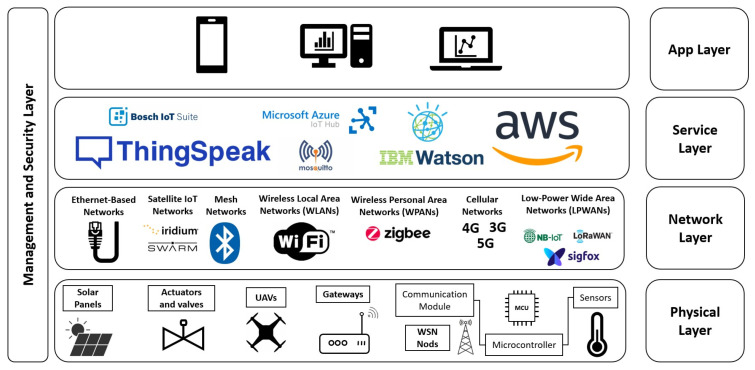
The typical layering architecture of an IoT device using relevant examples.

IoT encompass a wide array of sensors, drones, actuators, smart vehicles, and communication networks that generate, transmit, and analyze data for better decision-making. The architecture of IoT systems in agriculture is typically divided into several layers (Figure 1), each playing a crucial role in enhancing agricultural processes and providing structured functionality [2]. The physical layer comprises sensors, actuators, drones, smart vehicles, and communication gateways. This layer collects real-time data on environmental parameters such as soil moisture, temperature, humidity, and pH. It also automates processes like irrigation and fertilization, utilizing communication technologies such as LoRa, Zigbee, Wi-Fi, Bluetooth, and cellular networks [9].

The network layer ensures reliable data transmission from remote fields to cloud computing platforms [10]. It supports communication protocols, security authentication, and long-range, low-power networks like LoRa, which are ideal for rural agricultural settings. On the other hand, the service support layer provides essential services to process and store data and enable IoT applications [11]. The application layer delivers user-friendly platforms for farmers, including precision farming tools, smart greenhouse-management systems, irrigation optimization, and supply-chain tracking [12]. These services can be accessed remotely via mobile applications. Finally, the management and security layer ensures system configuration, resource optimization, and cybersecurity through encryption, authentication, and intrusion detection, protecting sensitive agricultural data from cyber threats [13].

In recent years, the rapid development of the Internet of Things (IoT) and machine learning (ML) has revolutionized precision irrigation management, enabling real-time monitoring, data-driven decision-making, and optimized water use efficiency [14,15]. IoT-based wireless sensor networks (WSNs) facilitate continuous soil moisture, weather, and crop health monitoring, while ML algorithms analyze these datasets to predict irrigation demands and automate control systems [16,17]. Such advancements mitigate water scarcity challenges by reducing over-irrigation and improving crop yields [18,19], underscoring their transformative potential in sustainable agriculture.

Bibliometric analyses offer a powerful approach to achieve this aim by mapping the intellectual landscape, revealing influential research clusters, and identifying emerging themes and innovation hotspots. The following section synthesizes the main findings of previous bibliometric studies on IoT applications in agriculture, offering valuable insights into the current research landscape and guiding future innovation. Numerous bibliometric studies have explored its evolution, research trends, and thematic developments, providing valuable insights into the advancements and research trajectories of this transformative field.

One of the earliest comprehensive bibliometric analyses was conducted by Suebsombut et al. [20], which classified trends and future directions in smart-farming research. The study provided foundational insights into the early development of IoT applications in agriculture. Research priorities that focused on sensor networks, data analytics, and the early adoption of automation technologies were identified. Bouzembrak et al. [21] extended this early exploration by focusing on IoT applications in food safety. The bibliometric analysis highlighted the potential of IoT technologies for enhancing food safety monitoring and traceability throughout the agricultural supply chain. The authors identified emerging research trends centered on real-time monitoring systems and predictive analytics for contamination detection. The findings underscored the role of IoT in ensuring food security and consumer safety. Singh et al. [22] conducted a comprehensive bibliometric study that revealed the growing adoption of IoT technologies for agricultural operations, particularly in areas such as smart irrigation and precision farming. The study mapped key research themes, including the integration of IoT with machine learning and cloud computing for advanced data processing and decision-making. This work highlighted the increasing sophistication of IoT applications in agriculture, driven by the need for more efficient resource management. The authors proposed a research agenda for advancing Agriculture 4.0, which included the development of interoperable platforms and the adoption of advanced analytics for predictive modeling.

One of the early studies focusing specifically on IoT trends in irrigation management is [23], which provided an overview of IoT and irrigation approaches through bibliometric analysis. The findings indicated a strong focus on developing efficient irrigation systems that leverage IoT technologies for real-time monitoring and control. The study emphasized the role of IoT in mitigating the impacts of climate change on agricultural water resources. Starting from 2023, bibliometric analysis for IoT integration in agriculture provided a deeper thematic focus on specific applications of IoT in agriculture. Gurmessa and Assefa [24] conducted a scientometric analysis focusing on smart irrigation literature. Their findings indicated a growing emphasis on integrating IoT and sensor technologies for efficient water management. Key research gaps were identified, such as the need for standardized protocols and scalable solutions for IoT-enabled irrigation systems. Some of the interesting proposed future research directions were the development of low-cost sensors and the integration of renewable energy sources for powering IoT devices.

Ahmed et al. [25] focused on smart irrigation management for improving water productivity under climate change conditions in drylands. The study identified emerging research themes, such as drought prediction and adaptive irrigation strategies, which are critical for sustaining agricultural productivity in arid and semi-arid regions. Still on irrigation management, Abdullahi et al. [26] provided a data-driven bibliometric review on precision irrigation. The authors highlighted the critical role of IoT in enabling data-driven irrigation management practices. The analysis identified key research trends, such as the integration of remote-sensing technologies and the use of big data analytics for optimizing irrigation schedules. They emphasized the need for collaborative research efforts to develop scalable and cost-effective precision irrigation solutions. Also, Pang et al. [27] examined trends in smart irrigation for smart agriculture through their bibliometric analysis. Their study identified key research themes, such as sensor technologies, data analytics, and IoT-enabled automation. The authors emphasized the need for collaborative research efforts to address the challenges associated with implementing smart irrigation systems at scale.

On the other hand, Chen et al. [28] explored the application of IoT technologies in green stormwater infrastructure (GSI). Although primarily focused on urban environments, their study offered valuable insights into the potential of IoT for managing water resources in agricultural landscapes. The authors highlighted the need for cross-disciplinary research to harness the full potential of IoT in GSI and other water-management applications. Abdullahi, Mahmud, Abi Hassan, and Ali [26] traced the evolution of IoT applications in smart agriculture through a comprehensive bibliometric analysis. The study revealed a steady increase in research outputs and identified influential publications and collaboration networks. The authors highlighted the importance of IoT in enabling precision farming practices and improving resource management. They also identified key research trends, such as the integration of IoT with blockchain for enhanced data security and traceability.

In 2024, more specialized applications were targeted. Addow and Jimale [29] examined the integration of IoT for water-quality monitoring in agricultural systems. Their study highlighted the increasing interest in leveraging IoT technologies to monitor and improve water quality. Key research clusters were identified, centered around water-quality monitoring, sensor technologies, and data analytics, underscoring the role of IoT in achieving sustainable water management.

Yet, the dominance of IoT applications for smart irrigation continued. Sadiq et al. [30] presented a bibliometric review on IoT-enabled smart irrigation systems. Their analysis revealed a significant rise in research activities aimed at optimizing water use efficiency through real-time data collection and automated irrigation systems. The authors mapped key research themes and identified influential authors and institutions contributing to advancements in smart irrigation technologies. Sumarsono, Muflihah, and Afiatna [11] explored the broader landscape of IoT research in agriculture through their bibliometric analysis. They identified the main research hotspots and trends, noting a growing emphasis on precision agriculture, data-driven decision-making, and sensor networks. The study also highlighted emerging challenges, such as data privacy and the need for standardized protocols.

The adoption of IoT in agriculture has seen a dramatic increase since 2015, with a notable surge in research output from 2017 onwards [26,31]. This growth is largely attributed to the convergence of IoT with AI and machine learning (ML), which has enabled more sophisticated applications in smart agriculture. For instance, IoT technologies are now widely used for real-time monitoring of soil moisture, climate conditions, and crop health, enabling precision agriculture practices such as targeted irrigation and fertilization [27,32]. These applications have proven particularly valuable in optimizing resource use and improving crop yields, especially in water-scarce regions [25].

The use of IoT in precision agriculture has also been bolstered by the integration of emerging technologies such as unmanned aerial vehicles (UAVs) and hyperspectral imaging, which provide high-resolution data for crop monitoring and management [32]. The convergence of these technologies has enabled farmers to make more informed decisions, reduce input costs, and improve overall agricultural productivity. The following (Table 1) lists the main found bibliometric studies regarding IoT in the field of agriculture in recent years.

Some of the common key findings from the analysis of the reviewed previous bibliometric studies reveal that journals such as Sensors (Switzerland), IEEE, and Computers and Electronics in Agriculture are the most influential in this field [11,26]. Key authors and institutions from India and China have significantly contributed to the growth of IoT in agriculture [22,36]. The most-cited topics include precision agriculture, smart farming, and IoT-enabled irrigation systems, reflecting the field’s focus on sustainability and efficiency [27,32].

The reviewed bibliometric studies provided a comprehensive overview of the research landscape surrounding IoT applications in agriculture. They highlight the growing importance of IoT in enabling data-driven decision-making, optimizing resource management, and enhancing the sustainability and resilience of agricultural systems. Thus, as the volume of research in this field grows rapidly, it becomes increasingly important to systematically analyze and map the existing body of knowledge to identify trends, gaps, and future opportunities. A bibliometric analysis provides a quantitative and objective framework to evaluate the evolution of research, highlight influential contributions, and uncover the most prevalent technologies, platforms, and applications.

Within this context, this study aims at offering a comprehensive overview of the current state of IoT for smart irrigation by analyzing the existing literature, to identify key trends, and addressing specific research questions, including the most commonly used technologies, microcontrollers (MCUs) and the main applications of IoT in agriculture, and the evolution of research themes over time. Through this analysis, the study aims to provide a holistic understanding of the current state of IoT in agriculture, highlight emerging areas of interest, and offer insights for future research directions to advance the integration of IoT technologies in agricultural irrigation practices. Furthermore, it aims to serve as a resource for researchers, practitioners, and policymakers to guide future innovations and investments in this domain.

## 2. Methodology

To conduct a comprehensive review of the integration of Internet of Things (IoT) technologies in irrigation management, a two-step methodological approach was adopted, combining bibliometric analysis and systematic review.

First, a bibliometric analysis was conducted to map the research landscape, identify key trends, prolific authors, and influential institutions in the field. The Web of Science (WoS) database was used as the primary source, accessed on 20 December 2024, covering the period from January 2014 to December 2024 (10 years). A structured search query was applied using the following Boolean terms:

(“Internet of Things” OR “IoT” OR “Wireless sensing”) AND (“Irrigation Management” OR “Irrigation Scheduling” OR “Smart Irrigation”). This initial search retrieved 290 papers, forming the dataset for bibliometric analysis.

Following the bibliometric assessment, a systematic review was performed to provide an in-depth examination of IoT applications in smart irrigation.

The research questions included the following:

- What are the main applications of IoT-based smart irrigation systems?
- What are the most-used communication technologies in the analyzed sample?
- What are the most-used microcontrollers in the IoT-based smart irrigation systems in the analyzed sample?

**Figure 2:**
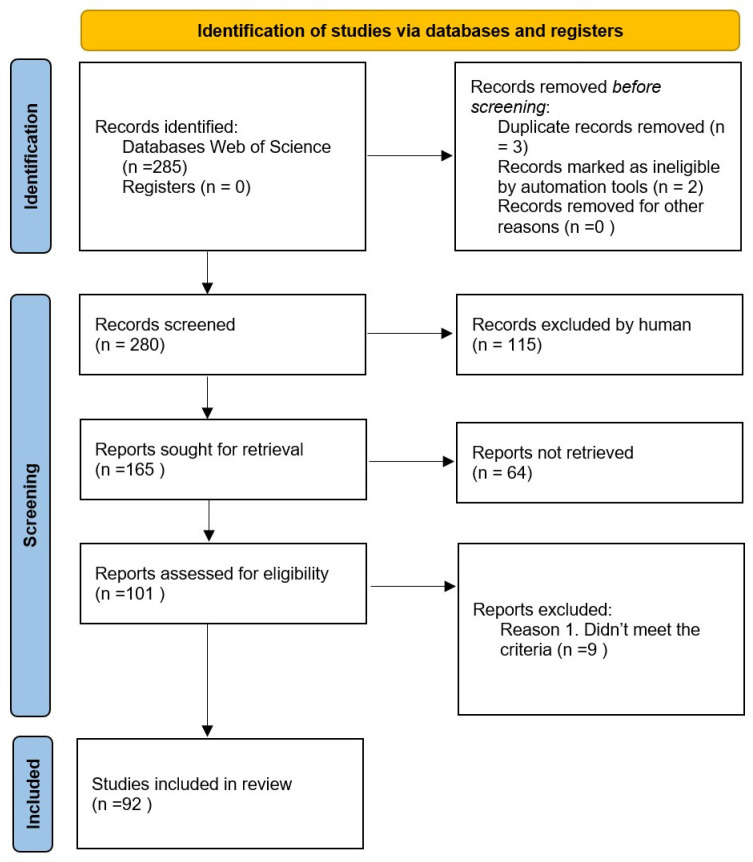
The screening process PRISMA chart.

To ensure a rigorous selection process, the SPIDER framework (Sample, Phenomenon of Interest, Design, Evaluation, Research Type) [42] was applied to establish inclusion and exclusion criteria (Table 2). This step enabled the identification of relevant studies based on their focus on IoT-driven irrigation management, technological advancements, and decision-support frameworks. After applying the screening process, a refined subset of 92 studies was selected for qualitative analysis [43]. Figure 2 shows the PRISMA chart [44] followed in the screening process.

VOSviewer [45] was used for network visualization and co-occurrence mapping of the bibliometric analysis, allowing the identification of key research clusters, influential authors, and collaborative networks. It employs the following steps:

(I) Co-occurrence and similarity calculation using similarity scores between entities (authors, journals, keywords, documents). This is calculated based on three bibliographic relationships:
  (a) Co-authorship analysis for authors, organizations, or countries where the co-authorship strength $S_{ij}$ between two authors i and j is defined as [46]
    (1) $$S_{ij}^{CA}=\frac{C_{ij}}{\sqrt{C_{i}C_{j}}}$$
    where $S_{ij}^{CA}$ represents the number of publications co-authored by i and j, and $C_{i}$ and $C_{j}$ denote the total number of publications by i and j, respectively.
  (b) Co-occurrence analysis of the keywords where the normalized co-occurrence similarity $S_{ij}$ is given by Equation (2) [47]:
    (2) $$S_{ij}^{kw}=\frac{C_{ij}}{C_{i}C_{j}}$$
    where $S_{ij}^{kw}$ represent the number of times keywords i and j appeared together in a publication, and $C_{i}$ and $C_{j}$ denote the total number of occurrences in publications of i and j, respectively.
  (c) Co-citation analysis for the sources using the co-citation strength $S_{ij}$ as determined by Equation (3):
    (3) $$S_{ij}^{CC}=\frac{{CC}_{ij}}{\sqrt{{CC}_{i}{CC}_{j}}}$$
    where ${CC}_{ij}$ represents the number of times documents i and j are cited together, and ${CC}_{i}$ and ${CC}_{j}$ denote the total citations received by documents i and j.
(II) Applying thresholds to filter and retain only the most relevant entities and connections using the minimum occurrences threshold, which ensures that only entities appearing at least N times are included. In addition to the minimum link strength threshold which is used to retain only strong connections by filtering based on similarity scores. VOSviewer adjusts this threshold dynamically based on the user’s input, dataset’s density and visual clarity. An entity is included if
  $$C_{i}\geq N$$
  or
  $$S_{i}\geq min.link\;strength\;threshold$$
  where $C_{i}$ represents the occurrences of entity i, and $S_{i}$ is the similarity/link strength.
(III) Optimizing the visualization: the VOS (visualization of similarities) mapping technique is applied, which minimizes the following objective function (Equation (4)) [48]:
  (4) $$\sum_{i<j}S_{ij}\left(d_{ij}-1\right)\left(d_{ij}-1\right)$$
  where $d_{ij}$ is the Euclidean distance between items I and j, and $S_{ij}$ represents the similarity strength. This function ensures that similar items are positioned closer together while maintaining clarity in the visualization.
(IV) Grouping similar entities (clustering): to detect meaningful groups, clustering using a modularity-based approach is applied (Equation (5)) [49,50].
  (5) $$Q=\frac{1}{2m}\sum_{ij}(A_{ij}-\frac{K_{i}K_{j}}{2m})\delta (C_{i}{,C}_{j})$$
  where $A_{ij}$ represents the actual weight of the link between i and j, $K_{i}$ is the sum of the weights for node i, m is the total weight of all links, and $\delta (C_{i}{,C}_{j})$) equals 1 if i and j belong to the same cluster; otherwise, 0.

Yet, the analysis was limited to the WoS database, potentially excluding relevant studies indexed in other databases. It is also worth mentioning that bibliometric tools rely on metadata quality; any inconsistencies in the WoS database could affect the analysis.

## 3. Results

### 3.1. Yearly Publication Count

**Figure 3:**
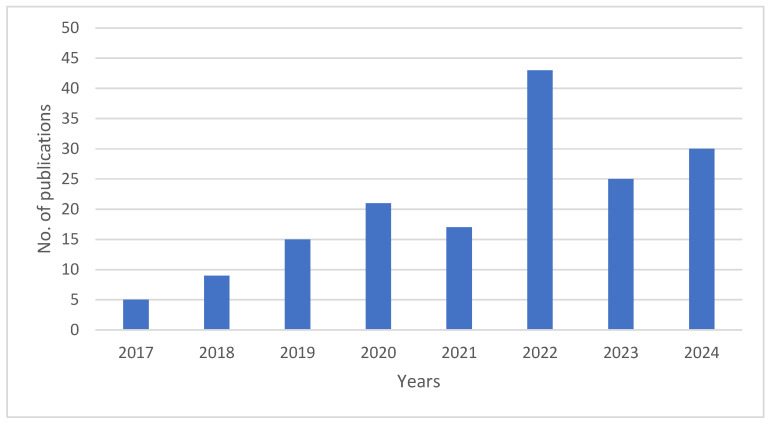
Trends in IoT in irrigation management.

Based on the publication trends of IoT in irrigation management from 2017 to 2024 (Figure 3), several key observations can be made. Between 2017 and 2019, the number of publications remained relatively low, with a gradual increase from five articles in 2017 to fourteen in 2019. This period represents an early phase in the research domain, where interest was emerging but had not yet gained significant traction.

From 2020 to 2022, the number of publications saw a steady increase, reaching 21 in 2020 and peaking at 45 in 2022. This substantial growth suggests an increasing scholarly interest in applying IoT technologies to irrigation management, likely driven by advancements in sensor networks, data analytics, automated irrigation systems, and the cost effectiveness of solid-state sensors and open-source hardware. The rise in publications during this period aligns with the broader trend of digital transformation in agriculture, where IoT is increasingly recognized as a key enabler of precision irrigation.

However, in 2023, the number of publications dropped to 25, followed by a slight increase to 31 in 2024. This decline in 2023, despite the previous growth trend, may indicate a shift towards more applied and interdisciplinary studies integrating IoT with other smart agriculture technologies. The rebound in 2024 suggests a renewed focus on IoT in irrigation management, possibly reflecting emerging challenges such as climate change adaptation, water resource optimization, integration with AI-driven decision support systems, and the emergence of low-cost solid-state sensors. The data underscore that IoT for irrigation management has evolved from a niche research area to a recognized field of study. While the number of publications fluctuated in recent years, the overall trend indicates growing interest and continued advancements in leveraging IoT for sustainable and efficient water management in agriculture.

### 3.2. Co-Authorship Collaboration Among Countries

**Figure 4:**
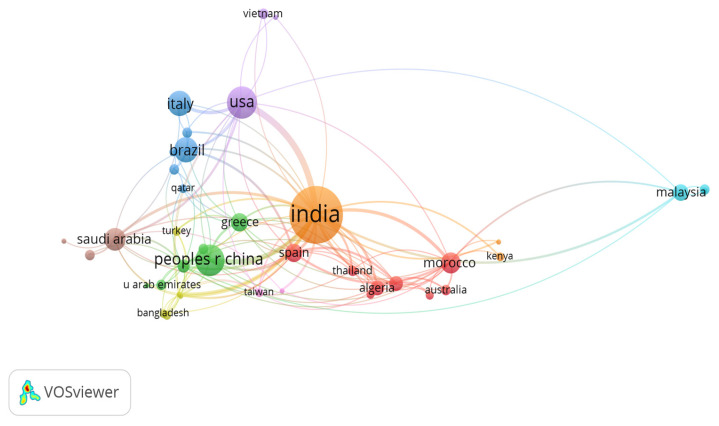
Co-authorship network map based on collaborations between countries.

As illustrated in Figure 4, an analysis of collaborative authorship in the field of IoT applications for irrigation management indicates that the most active contributing countries are India, China, the United States, Brazil, Italy, and Saudi Arabia. This distribution aligns with global trends in agricultural research, where both emerging and established economies play key roles in advancing smart irrigation technologies.

India and China exhibit significant research activity in IoT-driven irrigation management, reflecting their large agricultural sectors and increasing pressures related to water scarcity and food security. The substantial number of collaborative publications from these nations suggests a strong commitment to integrating digital technologies for enhancing agricultural productivity and optimizing water resources. Notably, China’s extensive investments in smart agriculture and IoT infrastructure have fostered robust academic and industrial collaborations. The United States, a leader in agricultural innovation, remains a dominant contributor to research and development in IoT-based irrigation. With well-established research institutions and government-supported precision agriculture initiatives, the U.S. is at the forefront of advancements in sensor technologies, machine learning applications, and decision-support systems for optimized water management. The high level of international collaborations involving U.S.-based researchers highlights efforts to disseminate technological advancements to regions facing similar agricultural challenges. Brazil’s strong presence among the top contributors underscores its increasing focus on precision irrigation, particularly given its expansive agricultural landscapes that rely heavily on water-intensive crops. As one of the world’s leading food producers, Brazil’s engagement in collaborative research on IoT applications in irrigation reflects a strategic initiative to improve resource efficiency and mitigate climate-induced variability in agricultural productivity. Italy, despite its relatively smaller geographic and population size, remains a significant contributor to scientific research on IoT-driven irrigation systems. The country’s well-established tradition of agricultural innovation, particularly within Mediterranean cropping systems where water management is critical, has driven interdisciplinary collaborations. Italian research institutions frequently integrate IoT technologies with agronomy, hydrology, and environmental sciences to develop advanced irrigation solutions. Saudi Arabia’s inclusion among the leading contributors reflects its growing investment in sustainable agricultural practices, particularly in arid and semi-arid regions where water conservation is a national priority. Given the country’s heavy reliance on irrigation for food production, there has been a concerted effort to adopt smart irrigation systems powered by IoT to enhance water-use efficiency. The high level of international collaboration involving Saudi researchers indicates ongoing efforts to adapt and implement digital irrigation solutions tailored to desert agriculture. A closer look at international collaborations highlights the increasingly interconnected nature of research in IoT-driven irrigation management. The exchange of knowledge between water-scarce regions and technologically advanced research hubs demonstrates a concerted effort to address global agricultural water challenges through innovative digital solutions. These findings emphasize the importance of cross-border partnerships in accelerating the adoption of smart irrigation technologies and ensuring their successful implementation across diverse agro-climatic regions.

**Figure 5:**
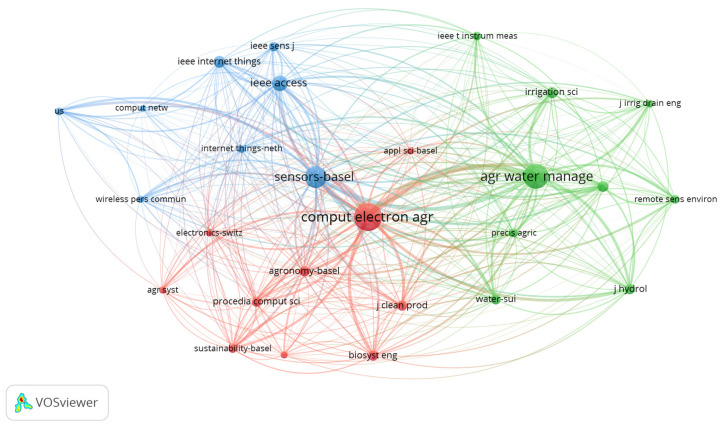
Co-citation network map based on scientific journals.

The bibliometric analysis of the most cited journals in IoT-based smart irrigation (Figure 5) research reveals three clusters with a strong interdisciplinary connection between agriculture, water management, sensor technology, and IoT applications. The journal Computer Electronics and Agriculture stands out as the most influential source, with 388 citations and the highest total link strength of 8184, indicating its central role in disseminating research at the intersection of IoT and agricultural automation. Similarly, Agriculture Water Management (266 citations, 6035 link strength) underscores the critical role of water-use efficiency studies in smart irrigation systems. The prominence of Sensors—Basel (214 citations, 4980 link strength) highlights the growing focus on sensor technologies for precision agriculture and irrigation control. The presence of IEEE Access (110 citations) and IEEE Internet of Things (64 citations) in the top-ranking journals demonstrates the integration of advanced IoT frameworks, networking protocols, and real-time data analytics in irrigation research.

Journals related to hydrology (Journal of Hydrology, 46 citations), environmental monitoring (Remote Sensing, 52 citations), and sustainability (Sustainability—Basel, 36 citations) indicate that IoT in irrigation is closely linked to climate adaptation, remote sensing, and sustainable water resource management. Meanwhile, Precision Agriculture (37 citations, 1105 link strength) and Irrigation Science (54 citations, 1273 link strength) emphasize the impact of IoT on optimizing irrigation techniques.

The presence of IEEE Sensors (41 citations), Internet of Things (29 citations), and Wireless Personal Communications (23 citations) further highlights the technological aspect of IoT implementation in agriculture. This bibliometric analysis confirms that IoT-based irrigation research is highly interdisciplinary, combining agriculture, hydrology, sensor networks, and computing technologies to develop more efficient, data-driven irrigation solutions.

**Figure 6:**
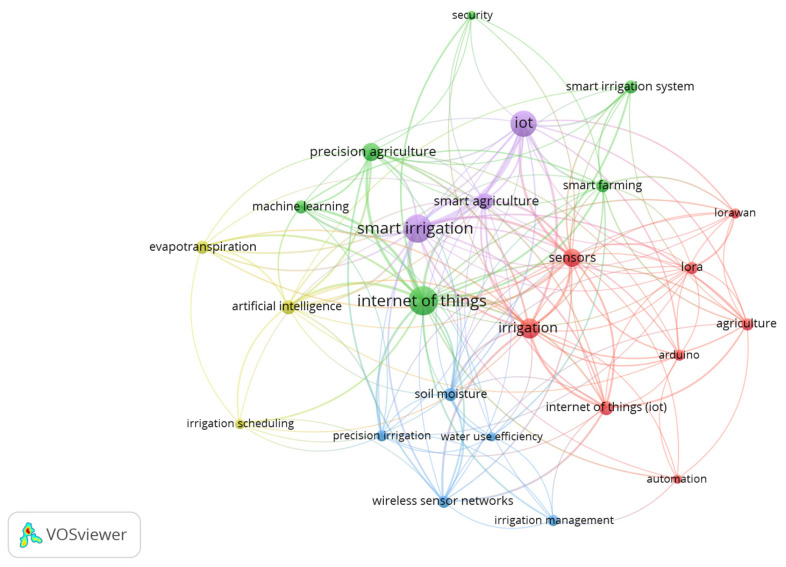
Co-occurrence network map based on authors’ keywords.

The analysis of the most relevant authors’ keywords on the topic (Figure 6) reveals the topics and applications; Internet of Things (49 occurrences and 251 link strength), smart irrigation (48 occurrences and 242 link strength), irrigation (25 occurrences and 166 link strength), sensors (20 occurrences and 134 link strength), precision agriculture (19 occurrences and 103 link strength), and wireless sensor networks (9 occurrences and 78 link strength) are high-frequency phrases in smart irrigation management research.

### 3.3. Microcontrollers and Boards

**Figure 7:**
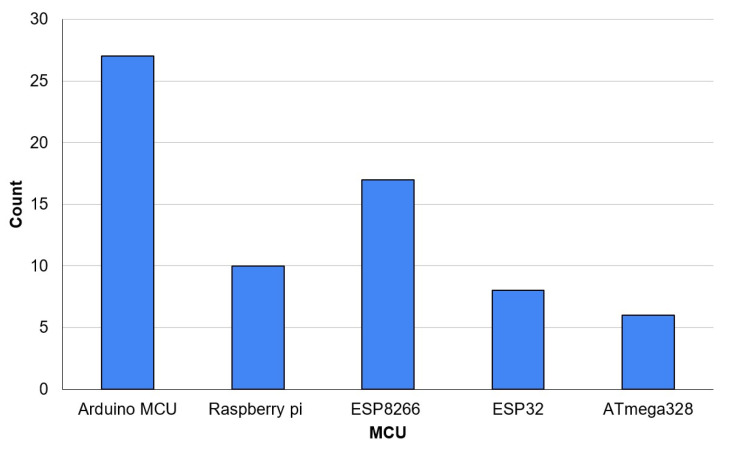
Highest-used MCUs in the analyzed sample.

The selection of microcontrollers in IoT-based smart irrigation research is a crucial factor that directly influences the performance, scalability, and practicality of implemented solutions. Microcontrollers serve as the backbone of IoT systems, handling sensor data acquisition, processing, and communication with cloud platforms or edge computing nodes. Their efficiency in terms of power consumption, computational capability, and compatibility with communication protocols (such as LoRa, Wi-Fi, or Zigbee) determines the feasibility of deploying large-scale, low-maintenance irrigation networks. Identifying the most commonly used microcontrollers in scientific publications provides insight into prevailing technological preferences, highlighting trends in hardware selection based on cost, availability, ease of integration, and support for real-time data processing. Additionally, understanding the dominant microcontroller platforms can help guide future research toward optimizing firmware, enhancing interoperability between different IoT nodes, and improving power management strategies. Given the increasing need for precision irrigation in resource-scarce environments, selecting microcontrollers with low energy consumption and robust connectivity features is vital to ensuring sustainable and long-term adoption of IoT-driven irrigation systems. The analyzed sample revealed that Arduino UNO (Arduino LLC, Ivrea, Italy), Raspberry Pi (Raspberry Pi Foundation, Cambridge, UK), ESP8266 (Espressif Systems, Shanghai, China), ESP32 (Espressif Systems, Shanghai, China) and ATmega328P (Microchip Technology, Chandler, AZ, USA) were the most used MCUs in the research respectively. The analysis also showed that there are emerging IoT MCUs with high growth potential and market expansion of IoT MCUs providing diversity of options. Table 3 shows all the reported used microcontrollers in the analyzed sample, along with the publications using each MCU and their country of origin, while Figure 7 shows the most-used MCUs.

### 3.4. Communication Technologies

**Figure 8:**
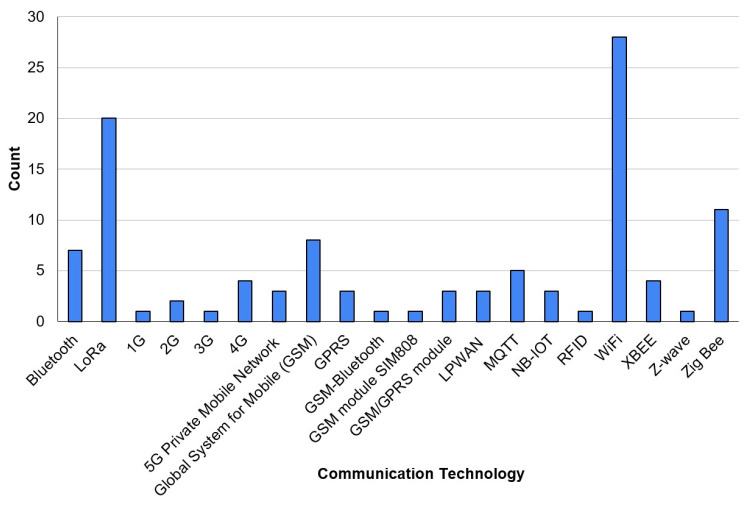
The most-used communication technologies.

The analysis of the scientific publications on IoT-based irrigation systems provides valuable insights into the most commonly used communication technologies (Figure 8). WiFi emerged as the most frequently cited technology, followed closely by LoRa and ZigBee. These figures indicate that while WiFi remains a dominant choice in current research, there is a noticeable shift toward LoRa and ZigBee, reflecting their increasing relevance in precision irrigation applications.

WiFi remains a widely used communication protocol for IoT-enabled irrigation due to its high-speed connectivity, ease of integration with cloud platforms, and existing infrastructure availability. However, its reliance on stable internet access, high power consumption, and limited transmission range (typically under 100 m in open fields) present significant challenges for large-scale agricultural applications. WiFi-based systems are often deployed in controlled environments, such as research stations, greenhouses, and small-scale farms, where network infrastructure is accessible, and power constraints are less significant.

Despite its advantages, the increasing focus on remote, autonomous, and energy-efficient irrigation systems has led researchers to explore alternative communication technologies. The growing number of publications on LoRa and ZigBee suggests that researchers are actively investigating these alternatives to address the limitations of WiFi-based smart irrigation networks. Unlike WiFi, which requires significant energy and infrastructure, LoRa operates over long distances (up to 10–15 km in rural areas) while consuming minimal power, making it ideal for monitoring soil moisture, weather conditions, and water distribution across expansive agricultural fields. Its key benefits in irrigation include the following:

- Extended coverage, enabling the deployment of sensors across large farms without needing extensive network infrastructure.
- Low power consumption, allowing battery-operated devices to function for several years with minimal maintenance.
- Cost-effectiveness, as LoRa operates in unlicensed frequency bands, reducing operational expenses compared to cellular networks.

Given these advantages, the near-equal number of publications on WiFi and LoRa suggests that LoRa is rapidly becoming a mainstream alternative, particularly in regions where power and internet infrastructure are unreliable.

ZigBee has also gained considerable attention in smart-irrigation research, particularly in studies conducted in greenhouses, orchards, and smallholder farms where multiple sensors must communicate efficiently. Unlike LoRa, which excels in long-range communication, ZigBee is designed for short-range, low-power, and low-data-rate applications, making it an ideal choice for localized irrigation management systems.

While ZigBee has fewer publications than WiFi and LoRa, its significant presence underscores its importance in scenarios where precise, localized control is required. Researchers have investigated ZigBee’s potential in automated irrigation scheduling, soil moisture sensing, and climate control within protected agricultural environments.

Given this shift, future research and practical implementations of IoT-based irrigation should prioritize energy-efficient, scalable, and long-range communication technologies. The increasing focus on LoRa and ZigBee in scientific literature indicates their growing adoption in real-world agricultural applications, paving the way for more sustainable and intelligent irrigation management solutions.

### 3.5. The Main Objective of IoT Sensing in Irrigation Systems

**Figure 9:**
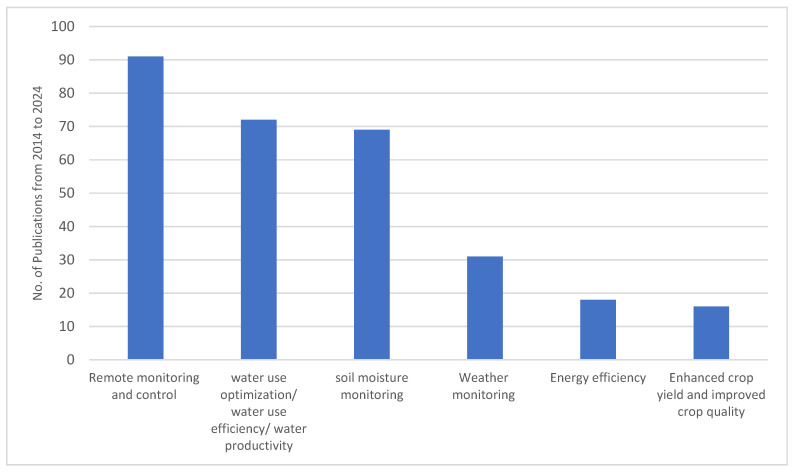
Main applications of IoT in smart irrigation as reported from 2014 to 2024.

The most prevalent applications included remote monitoring and control, water use optimization, soil moisture monitoring, weather monitoring, energy efficiency, and crop yield improvement, as shown in Figure 9. These findings highlight the critical areas where IoT solutions are being leveraged to enhance irrigation management. Below, we provide a detailed discussion of each of these trends and their implications.

The most frequently reported IoT application in smart irrigation is remote monitoring and control, with 91 publications emphasizing this capability. Remote monitoring enables farmers and irrigation managers to oversee their irrigation systems in real time, reducing the need for manual field visits and ensuring timely responses to changes in soil moisture levels, weather conditions, and crop water requirements [102]. IoT-based remote control systems allow for automated adjustments to irrigation schedules, valve operations, and pump activation based on real-time data.

This trend suggests that researchers and practitioners recognize the importance of remote accessibility in improving irrigation efficiency and reducing labor costs. The widespread adoption of remote monitoring and control is particularly beneficial in regions with limited agricultural labor availability or where large-scale farming operations make manual irrigation management impractical [103]. Additionally, real-time monitoring can help prevent water wastage by detecting leaks, clogged emitters, or system malfunctions, ultimately contributing to water conservation efforts.

The second-most reported IoT application is water use optimization, with 72 publications focusing on enhancing water efficiency and productivity. Efficient water management is a primary goal of smart irrigation, as excessive or insufficient irrigation can negatively impact crop health, soil quality, and overall agricultural sustainability.

IoT-enabled irrigation systems optimize water use by integrating data from soil moisture sensors, weather forecasts, and crop water requirements to provide precise irrigation recommendations. These systems help mitigate the risks of over-irrigation, which can lead to soil degradation and nutrient leaching, and under-irrigation, which can cause water stress and yield reduction. By applying only the required amount of water at the right time, IoT solutions contribute to sustainable water resource management and climate resilience in agriculture [104].

Soil moisture monitoring is another key area of IoT application, with 69 publications highlighting its significance in smart irrigation. Measuring soil moisture levels provides essential information for determining when and how much to irrigate [5,30,105]. IoT-based soil moisture sensors offer continuous real-time data, enabling precision irrigation strategies that align with crop needs and soil characteristics.

Accurate soil moisture monitoring prevents inefficient irrigation practices, such as watering when the soil already has adequate moisture or delaying irrigation until crops experience water stress. Moreover, integrating soil moisture data with remote sensing technologies and machine learning models enhances decision-making, allowing for adaptive irrigation strategies that respond dynamically to environmental conditions [106,107,108].

Weather monitoring plays a critical role in smart irrigation, as weather patterns directly influence evapotranspiration rates, soil moisture levels, and crop water requirements. Our research found that 31 publications discuss IoT applications in weather monitoring for irrigation management. IoT-based weather stations and connected meteorological sensors provide real-time data on temperature, humidity, wind speed, and solar radiation, which are used to predict irrigation needs more accurately.

Integrating weather data into irrigation decision-making helps optimize water use by aligning irrigation schedules with rainfall events, minimizing unnecessary watering, and preventing over-reliance on groundwater resources [25,32,109,110]. Additionally, advanced weather forecasting models powered by IoT data enable early warnings for drought conditions, allowing farmers to implement water conservation measures and adjust irrigation strategies accordingly.

Energy efficiency is a growing concern in smart irrigation, with 18 publications addressing its importance. Traditional irrigation methods often rely on energy-intensive pumping systems that contribute to high operational costs and environmental footprints [111]. IoT solutions improve energy efficiency by optimizing pump usage, reducing the frequency of manual interventions, and integrating renewable energy sources such as solar-powered irrigation systems [112,113].

By implementing smart energy-management strategies, IoT-enabled irrigation systems can significantly lower energy consumption while maintaining optimal irrigation performance. For example, variable frequency drive (VFD) controllers can adjust pump speeds based on real-time demand, preventing energy waste and extending equipment lifespan [114,115]. Additionally, IoT data analytics can identify inefficiencies in irrigation infrastructure, enabling proactive maintenance and reducing energy-intensive system failures [116,117].

While most IoT applications in irrigation focus on resource efficiency, 16 publications emphasize their role in enhancing crop yield and improving crop quality. Precision irrigation ensures that crops receive the right amount of water at critical growth stages, preventing drought stress and promoting uniform development. IoT-driven nutrient and water management strategies further enhance soil health, leading to improved crop nutrition and resilience against pests and diseases.

By integrating IoT-based irrigation with precision agriculture technologies such as drone imaging, spectral analysis, and artificial intelligence, farmers can optimize fertilizer application, detect plant health issues early, and enhance overall farm productivity. As global food demand continues to rise, leveraging IoT to maximize crop yield and quality will be instrumental in ensuring food security and agricultural sustainability.

The findings of this study highlight the transformative impact of IoT on modern irrigation practices. The emphasis on remote monitoring and control reflects a shift towards digital agriculture, where real-time data-driven decision-making is becoming the norm. The strong focus on water use optimization underscores the urgency of addressing water scarcity challenges and improving agricultural resilience against climate variability.

Moreover, the integration of soil moisture monitoring and weather data into irrigation management indicates an increasing reliance on predictive analytics for sustainable water resource management. Energy efficiency considerations suggest that smart irrigation technologies must not only optimize water use but also minimize environmental and financial costs associated with irrigation operations.

The relatively lower number of studies focusing on crop yield and quality improvement suggests that while resource efficiency remains a priority, more research is needed to explore the direct agronomic benefits of IoT-based irrigation. Future research should investigate how IoT applications influence crop physiology, nutrient uptake, and soil microbiome interactions to develop comprehensive irrigation solutions that maximize both water efficiency and agricultural productivity.

## 4. Challenges and Future Direction

The integration of the Internet of Things (IoT) in irrigation management presents a transformative approach to optimizing water use efficiency, enhancing crop productivity, and mitigating the impact of climate change on agriculture. While significant progress has been made in developing and deploying IoT-driven smart irrigation systems, from the reviewed sample, several key challenges hinder the large-scale adoption and long-term sustainability of IoT-based solutions. Addressing these challenges requires a holistic approach that considers technological, economic, environmental, and user-centered factors.

- Lack of universal standards: One of the most critical barriers to IoT adoption in irrigation management is the lack of universal standards for communication protocols, data exchange formats, and security frameworks [118]. The heterogeneity of IoT devices, manufactured by different vendors, often leads to compatibility issues, limiting the seamless integration of sensors, controllers, and cloud platforms [119]. Establishing stronger standardization practices would enhance interoperability, allowing farmers and water managers to adopt diverse IoT solutions without concerns about vendor lock-in. Furthermore, standardization would improve security across the entire IoT stack, ensuring that data transmitted from field sensors to cloud platforms remain protected against cyber threats and unauthorized access.
- Cybersecurity: Ensuring the security of IoT-based irrigation systems is paramount, as these systems rely on continuous data acquisition, transmission, and analysis to make real-time irrigation decisions. Current security strategies in agricultural IoT applications remain fragmented, with limited end-to-end encryption and physical security measures for field-deployed devices [120]. Developing robust security frameworks that incorporate encryption, blockchain-based data verification, and intrusion detection mechanisms would strengthen the resilience of IoT-enabled irrigation networks [121]. Moreover, raising awareness and providing training on cybersecurity best practices for farmers and agricultural stakeholders would further mitigate security risks.
- Modular-based solutions: A flexible and modular approach to designing IoT solutions for irrigation can significantly enhance their adoption and usability [14]. Modular hardware and software architectures enable easier customization, allowing farmers to tailor IoT deployments based on specific crop requirements, soil conditions, and water availability. Additionally, modularity supports scalability, ensuring that small-scale pilot deployments can be expanded into large-scale operations without requiring a complete system overhaul. By prioritizing modularity, developers can create IoT solutions that cater to both smallholder farmers and large commercial agricultural enterprises.
- Cost effectiveness: Despite the declining costs of embedded computing platforms, high-quality sensors and actuators remain expensive, limiting the affordability of IoT-based irrigation solutions for resource-constrained farmers. Reducing the overall unit cost of IoT hardware, internet connectivity, and data management services is essential for widespread adoption. This can be achieved through economies of scale in manufacturing, open-source development of sensor technologies, and government or private-sector subsidies that support precision irrigation initiatives. Furthermore, exploring innovative business models, such as pay-as-you-go or subscription-based IoT services, could make these technologies more accessible to farmers in developing regions.
- Compatibility with existing frameworks: The successful deployment of IoT solutions in irrigation management requires compatibility with existing agricultural infrastructure, including traditional irrigation systems, field machinery, and farm management software [15]. Developing IoT systems that can be retrofitted onto conventional irrigation networks—rather than requiring complete replacements—would encourage more farmers to transition toward digital water-management practices. This compatibility also extends to data integration, where IoT-based irrigation platforms should seamlessly interface with other agricultural decision-support systems, remote sensing data, and climate models to provide holistic insights for farm management.
- Robustness and reliability: IoT devices deployed in agricultural fields must withstand harsh environmental conditions, including extreme temperatures, humidity variations, and exposure to dust and chemicals [122]. Designing robust hardware that can endure seasonal and climate-related changes is essential to prevent frequent device failures and maintenance costs. Incorporating weatherproof enclosures, corrosion-resistant materials, and self-healing network architectures can enhance the durability of IoT irrigation systems, ensuring their reliable operation over extended periods [123].
- Studying the environmental impact of the resulting e-waste: As IoT adoption accelerates in agriculture, addressing the environmental impact of electronic waste from outdated or discarded devices becomes crucial. Sustainable practices, such as designing IoT hardware with recyclable materials, implementing take-back programs for old sensors, and promoting circular-economy principles, should be prioritized [25,124]. By integrating sustainability considerations into the design and deployment of IoT-based irrigation solutions, the agricultural sector can minimize its ecological footprint while maximizing the long-term benefits of precision irrigation.

The integration of IoT in irrigation management holds immense potential for enhancing water-use efficiency, optimizing crop growth, and mitigating climate-related agricultural challenges. However, several technical, economic, and operational barriers must be addressed to ensure widespread adoption. Standardization, power efficiency, security, scalability, cost reduction, and user-centered design are among the critical factors that will determine the success of IoT-enabled irrigation solutions. By fostering interdisciplinary research collaborations and leveraging advancements in sensor technology, data analytics, and machine learning, the future of smart irrigation can be both technologically robust and economically viable, ensuring sustainable agricultural water management for years to come.

Future research should focus on developing cost-effective IoT solutions, strengthening security frameworks, and enhancing user-friendly interfaces to promote mass adoption. Additionally, interdisciplinary collaborations between agronomists, engineers, and data scientists will be crucial in advancing IoT applications that address real-world agricultural challenges.

## 5. Conclusions

This systematic review provides a comprehensive overview of the research landscape surrounding the application of IoT in smart irrigation from 2014 to 2024. The analysis of publication trends indicates a significant rise in scholarly interest, with peak activity observed between 2020 and 2022, followed by a shift towards interdisciplinary and applied studies. Despite some fluctuations in publication numbers, the overall trajectory underscores the growing recognition of IoT as a transformative tool for precision irrigation and sustainable water management in agriculture.

The geographic distribution of contributions highlights the central role of countries such as India, China, and the United States in advancing IoT-based irrigation technologies. Their research efforts reflect diverse agricultural challenges, including water scarcity, food security, and climate adaptation, demonstrating the global relevance of IoT in optimizing irrigation practices. The strong international collaborations observed further reinforce the interconnected nature of agricultural research and the need for cross-border knowledge exchange to address common challenges.

The citation analysis of leading journals reveals that IoT in irrigation is inherently interdisciplinary, integrating agriculture, hydrology, sensor networks, and computing technologies. Highly cited journals such as Computers and Electronics in Agriculture, Agricultural Water Management, and Sensors—Basel serve as critical platforms for disseminating knowledge at the intersection of IoT and precision irrigation. The presence of journals focused on hydrology, environmental monitoring, and sustainability further indicates the broader implications of IoT in addressing water resource management and climate resilience.

Key technological trends in IoT-based smart irrigation, including the selection of microcontrollers and communication protocols, reveal a clear shift towards low-power, scalable, and cost-effective solutions. While WiFi remains a dominant communication technology, the increasing adoption of LoRa and ZigBee suggests a growing preference for energy-efficient and long-range communication in large-scale agricultural applications. The analysis of microcontrollers highlights the widespread use of Arduino, Raspberry Pi, ESP8266, and ESP32, indicating a preference for open-source, adaptable platforms suitable for real-time data acquisition and remote sensing.

The review of research applications underscores the primary objectives of IoT in irrigation systems, including remote monitoring and control, water use optimization, soil moisture and weather monitoring, energy efficiency, and crop yield improvement. The strong focus on real-time monitoring and automated irrigation control reflects a shift towards data-driven decision-making, reducing reliance on manual interventions and improving irrigation efficiency. The emphasis on energy efficiency and sustainability highlights the increasing need for smart irrigation technologies that minimize both water and energy consumption, ensuring long-term viability in resource-scarce environments.

Despite the extensive research on IoT applications in irrigation, certain gaps remain that warrant further investigation. While significant advancements have been made in sensor technologies, communication protocols, and automation, there is still a need to explore the agronomic impacts of IoT-based irrigation on crop physiology, nutrient dynamics, and soil health. Additionally, integrating IoT with emerging technologies such as artificial intelligence, edge computing, and blockchain could enhance decision-support systems, improve data security, and enable more adaptive and predictive irrigation strategies.

Overall, this systematic review confirms that IoT has evolved from an emerging concept to a well-established field of research in irrigation management. As global challenges related to water scarcity, food security, and climate change intensify, IoT-based smart irrigation systems will play a crucial role in enhancing agricultural resilience and sustainability. Future research should focus on refining existing technologies, fostering interdisciplinary collaborations, and developing scalable solutions that can be deployed across diverse agricultural landscapes to ensure efficient and sustainable water resource management in the years to come.

## Figures and Tables

**Table 1:** The recent bibliometric analysis regarding IoT in agriculture.

No	Article’s Title	Authors	Year
1	Integrating IoT and Water Quality: A Bibliometric Analysis	[29]	2024
2	Smart irrigation systems enabled with internet of things: a bibliometric review	[30]	2024
3	The Internet of Things Research in Agriculture: A Bibliometric Analysis	[11]	2024
4	Theme Mapping and Bibliometric Analysis of Two Decades of Smart Farming	[33]	2023
5	Artificial intelligence-based decision support systems in smart agriculture: Bibliometric analysis for operational insights and future directions	[31]	2023
6	A Bibliometric Analysis of the Evolution of IoT Applications in Smart Agriculture	[26]	2023
7	A Scoping Review of the Smart Irrigation Literature Using Scientometric Analysis	[24]	2023
8	Application of Internet of Things (IoT) Technologies in Green Stormwater Infrastructure (GSI): A Bibliometric Review	[28]	2023
9	Bibliometric Analysis of Trends in Smart Irrigation for Smart Agriculture	[27]	2023
10	Advancements in Smart Farming: A Comprehensive Review of IoT, Wireless Communication, Sensors, and Hardware for Agricultural Automation	[34]	2023
11	A data-driven bibliometric review on precision irrigation	[32]	2023
12	An Overview of Smart Irrigation Management for Improving Water Productivity under Climate Change in Drylands	[25]	2023
13	Review of artificial intelligence and internet of things technologies in land and water management research during 1991–2021: A bibliometric analysis	[35]	2023
14	The Interplay between the Internet of Things and agriculture: A bibliometric analysis and research agenda	[36]	2022
15	A Bibliometric Analysis and Review of Resource Management in Internet of Water Things: The Use of Game Theory	[37]	2022
16	An overview of the internet of things (IoT) and irrigation approach through bibliometric analysis	[23]	2021
17	Wireless Sensor Networks in Agriculture: Insights from Bibliometric Analysis	[38]	2021
18	The Digital Agricultural Revolution: A Bibliometric Analysis Literature Review	[39]	2021
19	Bibliometric Analysis of the Use of the Internet of Things in Precision Agriculture	[40]	2021
20	Trends on Advanced Information and Communication Technologies for Improving Agricultural Productivities: A Bibliometric Analysis	[1]	2020
21	Internet of things and agriculture relationship: a bibliometric analysis	[22]	2020
22	Internet of Things in food safety: Literature review and a bibliometric analysis	[21]	2019
23	A Bibliometric Analysis on Agriculture 4.0	[41]	2019
24	The using of bibliometric analysis to classify trends and future directions on “Smart Farm”	[20]	2017

**Table 2:** Applying SPIDER framework for inclusion and exclusion criteria.

Category	Definition	Inclusion Criteria	Exclusion Criteria
Sample (S)	The population of studies considered	Studies focusing on IoT-enabled irrigation management, smart irrigation systems, or wireless sensor networks in irrigation	Studies unrelated to IoT applications in irrigation or focusing on non-agricultural sectors
Phenomenon of Interest (PI)	The main topic investigated	IoT-based technological advancements in irrigation, including smart irrigation scheduling, decision-making frameworks, and water management	Studies without a focus on IoT, irrigation, or water management
Design (D)	Research design/methods used	Studies that provide technical details on IoT implementation in irrigation (e.g., sensor types, communication technologies, microcontrollers)	Opinion pieces, non-peer-reviewed articles, or studies lacking technical details
Evaluation (E)	The outcome measures or key findings analyzed	Studies presenting bibliometric insights (e.g., co-occurrence analysis, citation networks) and discussing key IoT components used in smart irrigation	Studies with incomplete metadata, inaccessible full texts, or minimal relevance to IoT-driven irrigation
Research type (R)	Type of research included	Empirical studies, systematic reviews, bibliometric analyses, and experimental studies on IoT in irrigation	Conference abstracts, patents, editorials, or studies outside the WoS database

**Table 3:** Listing all the reported MCUs in IoT for smart irrigation research between 2014 and 2024.

Board/MCU	Manufacturer	City	Country	References
Arduino Based MCUs	Arduino LLC	Ivrea	Italy	[51,52,53,54,55,56,57,58,59,60,61,62,63,64,65,66,67,68,69,70,71,72,73,74,75]
ATmega328 and ATmega328P	Microchip Technology	Chandler, Arizona	USA	[56,76,77,78,79,80]
ESP32	Espressif Systems	Shanghai	China	[5,8,81,82,83,84,85,86]
ESP8266	Espressif Systems	Shanghai	China	[51,53,58,60,61,63,76,77,83,87,88,89,90,91,92,93,94]
Raspberry Pi	Raspberry Pi Foundation	Cambridge	UK	[17,71,81,95,96,97,98,99,100,101]

## Data Availability

Data are contained within the article and Supplementary Materials.

## Supplementary Materials

The following supporting information can be downloaded at: https://www.mdpi.com/article/10.3390/s25072291/s1, Collected Publications Database.
