# Supplementary material for: IoT Sensing for Advanced Irrigation Management: A Systematic Review of Trends, Challenges, and Future Prospects
Source: Sensors (Basel). 2025 Apr 4;25(7):2291. doi: 10.3390/s25072291 (PMC11991392; doi:10.3390/s25072291)
Supplement: Supplementary file 1 [file sensors-25-02291-s001.zip › sensors-3504677 - PRISMA_2020_flow_diagram.docx]

**Identification of studies via databases and registers**

Records removed *before screening*:

Duplicate records removed (n = 3)

Records marked as ineligible by automation tools (n = 2)

Records removed for other reasons (n =0 )

Records identified from WoS:

Databases (n =290)

Registers (n = 0)

**Identification**

Records screened

(n = 285)

Records excluded by human**

(n = 116)

Reports sought for retrieval

(n =197 )

Reports not retrieved

(n = 28)

**Screening**

Reports assessed for eligibility

(n =169 )

Reports excluded:

Reason 1. Didn’t meet the criteria (n =8 )

Studies included in review

(n =165 )

**Included**

*Consider, if feasible to do so, reporting the number of records identified from each database or register searched (rather than the total number across all databases/registers).

**If automation tools were used, indicate how many records were excluded by a human and how many were excluded by automation tools.

Source: Page MJ, et al. BMJ 2021;372:n71. doi: 10.1136/bmj.n71.

This work is licensed under CC BY 4.0. To view a copy of this license, visit <https://creativecommons.org/licenses/by/4.0/>
