# Supplementary material for: IoT Sensing for Advanced Irrigation Management: A Systematic Review of Trends, Challenges, and Future Prospects
Source: Sensors (Basel). 2025 Apr 4;25(7):2291. doi: 10.3390/s25072291 (PMC11991392; doi:10.3390/s25072291)
Supplement: Supplementary file 1 [file sensors-25-02291-s001.zip › sensors-3504677 - PRISMA_2020_flow_diagram.pdf]

PRISMA 2020 flow diagram for new systematic reviews which included searches of databases and registers only

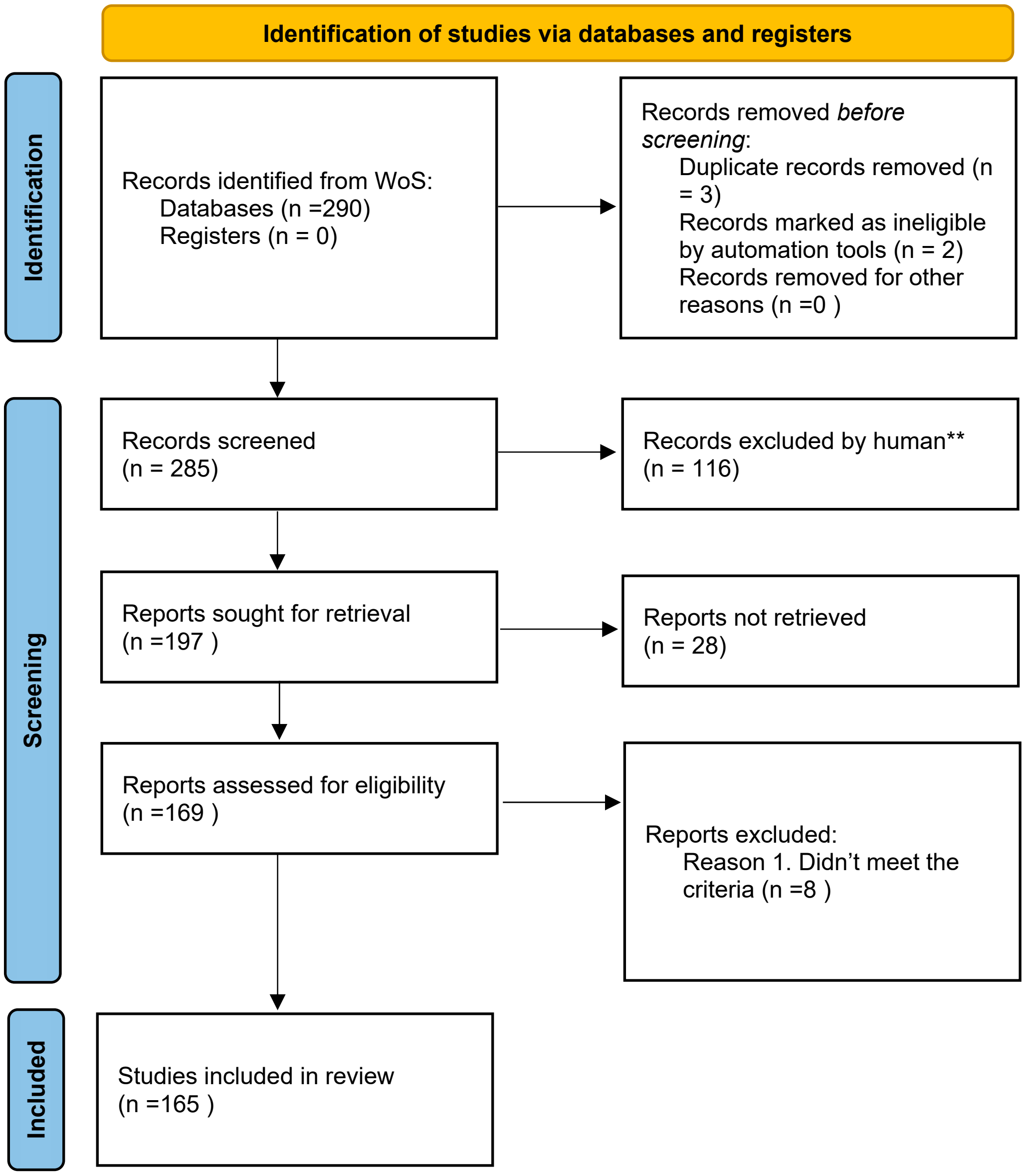

*Consider, if feasible to do so, reporting the number of records identified from each database or register searched (rather than the total number across all databases/registers).

**If automation tools were used, indicate how many records were excluded by a human and how many were excluded by automation tools.

Source: Page MJ, et al. BMJ 2021;372:n71. doi: 10.1136/bmj.n71.

This work is licensed under CC BY 4.0. To view a copy of this license, visit https://creativecommons.org/licenses/by/4.0/
